# Overexpression of RBM5 induces autophagy in human lung adenocarcinoma cells

**DOI:** 10.1186/s12957-016-0815-7

**Published:** 2016-02-29

**Authors:** Zhenzhong Su, Ke Wang, Ranwei Li, Jinzhi Yin, Yuqiu Hao, Xuejiao Lv, Junyao Li, Lijing Zhao, Yanwei Du, Ping Li, Jie Zhang

**Affiliations:** Department of Respiratory Medicine, The Second Affiliated Hospital of Jilin University, Changchun, Jilin 130041 People’s Republic of China; Department of Pathophysiology, Norman Bethune College of Medicine of Jilin University, Changchun, Jilin 130021 People’s Republic of China; Department of Paediatrics, The Second Affiliated Hospital of Jilin University, Chuangchun, Jilin 130041 People’s Republic of China; Department of Urinary Surgery, The Second Affiliated Hospital of Jilin University, Changchun, Jilin 130041 People’s Republic of China

**Keywords:** RNA binding motif 5, Autophagy, Non-small cell lung cancer, A549 xenograft, Gene expression

## Abstract

**Background:**

Dysfunctions in autophagy and apoptosis are closely interacted and play an important role in cancer development. RNA binding motif 5 (RBM5) is a tumor suppressor gene, which inhibits tumor cells’ growth and enhances chemosensitivity through inducing apoptosis in our previous studies. In this study, we investigated the relationship between RBM5 overexpression and autophagy in human lung adenocarcinoma cells.

**Methods:**

Human lung adenocarcinoma cancer (A549) cells were cultured in vitro and were transiently transfected with a RBM5 expressing plasmid (GV287-RBM5) or plasmid with scrambled control sequence. RBM5 expression was determined by semi-quantitative reverse transcription polymerase chain reaction (RT-PCR) and Western blot. Intracellular LC-3 I/II, Beclin-1, lysosome associated membrane protein-1 (LAMP1), Bcl-2, and NF-κB/p65 protein levels were detected by Western blot. Chemical staining with monodansylcadaverine (MDC) and acridine orange (AO) was applied to detect acidic vesicular organelles (AVOs). The ultrastructure changes were observed under transmission electron microscope (TEM). Then, transplanted tumor models of A549 cells on BALB/c nude mice were established and treated with the recombinant plasmids carried by attenuated Salmonella to induce RBM5 overexpression in tumor tissues. RBM5, LC-3, LAMP1, and Beclin1 expression was determined by immunohistochemistry staining in plasmids-treated A549 xenografts.

**Results:**

Our study demonstrated that overexpression of RBM5 caused an increase in the autophagy-related proteins including LC3-I, LC3-II, LC3-II/LC3-I ratio, Beclin1, and LAMP1 in A549 cells. A large number of autophagosomes with double-membrane structure and AVOs were detected in the cytoplasm of A549 cells transfected with GV287-RBM5 at 24 h. We observed that the protein level of NF-κB/P65 was increased and the protein level of Bcl-2 decreased by RBM5 overexpression. Furthermore, treatment with an autophagy inhibitor, 3-MA, enhanced RBM5-induced cell death and chemosensitivity in A549 cells. Furthermore, we successfully established the lung adenocarcinoma animal model using A549 cells. Overexpression of RBM5 enhanced the LC-3, LAMP1, and Beclin1 expression in the A549 xenografts.

**Conclusions:**

Our findings showed for the first time that RBM5 overexpression induced autophagy in human lung adenocarcinoma cells, which might be driven by upregulation of Beclin1, NF-κB/P65, and downregulation of Bcl-2. RBM5-enhanced autophagy acts in a cytoprotective way and inhibition of autophagy may improve the anti-tumor efficacy of RBM5 in lung cancer.

## Background

Lung cancer is the leading cause of cancer-related mortality in the world [1]. Nearly 85 % of these are non-small cell lung cancer (NSCLC), which typically has a poor prognosis with the overall 5-year survival rate of ~17 % despite innovations in diagnostic testing, surgical technique, and development of new chemotherapeutic agents [1, 2]. Thus, studies on new therapeutic strategies or targets are of great interest. Several of these studies are designed to target RBM5, since deregulation of RBM5 and its downstream targets have been linked to tumorigenesis and tumor maintenance in a variety of cancers, including lung cancer, breast cancer, pancreatic cancer, prostate cancer, and vestibular schwannoma [3–8].

RNA binding motif 5 (RBM5) (also called Luca15 or H37), the tumor suppressor gene, maps to the human chromosomal locus 3p21.3, which is strongly associated with lung cancer [9]. It has been shown to function as a regulator of apoptosis and cell cycle arrest in several malignancies, particularly in NSCLC cells through pre-messenger RNA (mRNA) splicing of multiple target genes [10–12]. The ectopic expression of RBM5 suppresses the growth of human lung cancer [11], breast cancer [13], fibrosarcoma [14], and hematopoietic cells [15–17]. Our previous study demonstrated that exogenous expression of RBM5 by the pcDNA3.1-RBM5 inhibited the cell growth of human prostate cancer and lung cancer in vivo and in vitro and resensitized the response of A549/DDP cells (cisplatin resistant counterparts of A549 cells) to cisplatin [8, 18–20]. Although the mechanisms of RBM5-mediated tumor suppression remain not quite clear, recent studies suggest that RBM5 is involved in the regulation of the mitochondrial apoptotic pathway and Bcl-2 family expression [8, 18, 19]. It has been confirmed that Bcl-2 family also function as autophagy regulators via their interaction with the core autophagy factor Beclin family which plays an important role in the initiation of autophagosome formation [21–24]. However, the relationship between RBM5 and autophagy in human lung cancer is still unknown.

Autophagy is an evolutionarily conserved process in all eukaryotic cells, which is used for recycling cytoplasm to generate macromolecular building blocks and energy under stress conditions, to remove superfluous and damaged proteins and organelles to adapt to changing nutrient conditions and to maintain cellular homeostasis [25, 26]. Autophagy has been recognized as having a complex impact on the initiation, progression, and treatment of lung cancer [27]. Low expression of Beclin1 was significantly associated with tumorigenesis, tumor progression, and poor prognosis in NSCLC [28]. Inactivation of the essential autophagy gene Atg5 causes an acceleration of the early phases of NSCLC oncogenesis [29]. Suppression of basal autophagy leads to inhibition of NSCLC cell proliferation and sensitizes them to cisplatin-induced caspase-dependent and caspase-independent apoptosis [30]. The initiation and execution of autophagy is regulated by a number of intracellular molecules, such as autophagy-related (Atg) proteins, mTORC1, AMPK, Beclin1, Bcl-2, and PI3K/AKT pathway [27]. Although the understanding of the regulation of autophagy has substantially advanced, information on the regulation of this complex process is limited.

In this study, we investigated the relationship of RBM5 overexpression and its potential role of autophagy modulation, which will help us to learn more about the mechanism of potential activity of RBM5 and autophagy regulation in NSCLC. Our data showed for the first time that RBM5 overexpression induced autophagy, which might be regulated by the alteration of NF-κB/P65 and Bcl-2 expression.

## Methods

### Cell lines and expression vectors

The human lung adenocarcinoma cell line A549 cells were obtained from the Chinese Academy of Medical Sciences. A549 cells were cultured in RPMI-1640 medium (Gibco, USA), supplemented with 10 % fetal bovine serum (Gibco, USA), 100 U/mL penicillin, and 100 μg/mL streptomycin (Sigma-Aldrich, St. Louis, USA) at 37 °C and 5 % CO_2_ with high humidity.

### Expression vectors and reagents

RBM5 expression plasmid (NM_005778) (GV287-RBM5) and the plasmid with scrambled control sequence (GV287) were constructed by Genechem Company (Genechem, China). Cisplatin were purchased from Sigma Chemical Co. (Sigma Chemical Co., St. Louis, USA).

### Transient transfections and treatments

A549 cells were transiently transfected with recombined plasmid with RBM5 gene (GV287-RBM5) or plasmid with scrambled control sequence using Lipofectamine 2000 reagent (Invitrogen, Carlsbad, CA) according to the manufacturer’s instruction as described previously [19, 20, 31], with 4 μg DNA for 9 × 10^5^ A549 cells in a six-well plate. Five hours after the transfection, the cell culture medium was replaced with 10 % fetal bovine serum. For the pharmacological modulation of autophagy, cells were treated with 3-MA (5 mM), which is a class III phosphatidylinositol 3-kinase (PtdIns3K) inhibitor and can block the early steps of autophagy [32] at the same time. Twenty-four hours after transfection, the cells were stained, or harvested, or treated with cisplatin for another 24 h for MTT assays.

### Cell viability assays

Cell viability was determined using 3-(4,5-dimethylthia-zol-2-yl)-2,5-diphenyltetrazolium bromide (MTT) assays. Cells were seeded at 2 × 10^4^ cells in 100 μl per well in 96-well plates for 12 h. Then, cells were treated with different concentrations of cisplatin for 24 h. The cells were treated with 0.5 mg/ml MTT solution and incubated for 4 h at 37 °C in the dark. The supernatant was aspirated, and formazan crystals were dissolved in 100 μl of DMSO at 37 °C for 15 min with gentle agitation. The absorbance value at a wavelength of 570 nm was measured with a microplate reader (Bio-Rad, Richmond, CA). Cell viability was calculated as a percentage of the control (untreated) values. All experiments were carried out in triplicate.

### Acridine orange staining

Acridine orange (AO) (Sigma Chemical Co., St. Louis, USA) was used to evaluate the formation of acid vesicular organelles (AVOs) by fluorescence microscopy. AO is a fluorescent molecule used to identify either apoptotic cell death or autophagy. It can interact with DNA emitting green fluorescence or accumulate in acidic organelles in which it becomes protonated forming aggregates that emit bright red fluorescence. After transfected with plasmids for 24 h, cells were treated with AO (1 μg/ml) in a serum-free medium and incubated for 15 min at 37 °C in the dark. Then, cells were washed four times with PBS, and fluorescent micrographs were obtained using an inverted fluorescence microscope (Olympus, Japan).

### MDC incorporation assay

Another fluorescent compound, monodansylcadaverine (MDC) has been proposed as a tracer for autophagic vacuoles. Autophagic vacuoles were also detected with MDC staining. After transfected with plasmids for 24 h, cells were incubated with MDC (50 μM) (Sigma Chemical Co., St. Louis, USA) in a serum-free medium at 37 °C for 30 min in the dark. After incubation, cells were washed four times with cold PBS, and fluorescent micrographs were obtained using an inverted fluorescence microscope (Olympus, Japan).

### Transmission electron microscopy

Treated cells were washed and fixed in 2.5 % glutaraldehyde for 2 h at 4 °C and then treated with 1 % osmium tetroxide, dehydrated in a graded series of ethanol baths, infiltrated and embedded in Epon resin. Ultrathin sections of 70 nM were cut in a Leica microtome (Leica, Deerfield, III), poststained with uranyl acetate and lead citrate, and examined in a HITACHI H-7650 transmission electron microscope (TEM) (HITACHI, Ltd, Japan) at an accelerating voltage of 80 kV.

### Protein extraction and Western blot

Total protein from cultured cells was extracted according to the previous study [31]. Briefly, protein concentration was measured by the Protein Assay Kit (Bio-Rad, CA). Equal amounts of protein samples (20–50 μg) were separated by 12 % SDS-PAGE and transferred onto poly (vinyl idene fluoride) (PVDF) membranes (Millipore, USA). The membranes were treated with Tris-buffered saline and Tween-20 solution (TBST) containing 50 g/L skim milk at room temperature for 1 h, and incubated overnight at 4 °C with an antibody against RBM5 (Santa Cruz Biotechnology, CA), LC3A/B (Proteintech Group, USA), Beclin1 (Proteintech Group, USA), Bcl-2 (Proteintech Group, USA), NF-κB/p65 (Affinity, USA), or LAMP1 (Abcam, USA). The mouse monoclonal antibody against β-actin (Proteintech Group, USA) was used as a housekeeping control gene. Membranes were washed three times for 10 min with TBST and incubated with horseradish peroxidase-conjugated secondary antibodies (Proteintech Group, USA) at a dilution of 1:500 for 1 h at room temperature. Membranes were washed three times for 10 min with TBST, and bands were then detected using an Enhanced Chemiluminescence (ECL) Detection kit (Pierce Biotechnology Ltd., Rockford, IL, USA) and quantified by densitometry using Quantity One software (Bio-Rad).

### RNA extraction and semi-quantitative RT-PCR

Reverse transcription-polymerase chain reaction (RT-PCR) was performed essentially as described previously [20]. In brief, total RNA was extracted from the cells using a Trizol reagent (Invitrogen, Carlsbad, CA) according to the manufacturer’s protocol. The ratio of absorbance at 260 and 280 nm (A260/280) was measured to assess RNA purity and quantity. First-strand complementary DNA (cDNA) was generated using All-in-One^™^ First-Strand cDNA Synthesis Kit (GeneCopoeia, Inc, USA) according to the manufacturer’s instructions. Primers were made by Sangon Biotech (Sangon Biotech, China). The primer sequences were as follows: GAPDH sense: 5′-CATGTAGTTGAGGTCAATGAAGG-3′ and antisense: 5′-GAGCCACATCGCTCAGACAC-3′; RBM5 sense: 5′-GCACGACTATAGGCATGACAT-3′ and antisense: 5′-AGTCAAACTTGTCTGCTCCA-3′. PCR was performed at 95 °C for 5 min and 28–30 cycles of 95 °C for 30 s, 58 °C for 30 s, 72 °C for 45 s, and 72 °C for 10 min. The PCR products were separated by electrophoresis on 1 % agarose gels containing ethidium bromide. The PCR products were visualized by a Tanon-1600 figure gel image processing system and analyzed with a GIS 1D gel image system software (Tanon, Shanghai, China).

### Establishment of A549 xenografts

The use of animal was in accordance with animal care guidelines, and the protocol was approved by Jilin University Animal Care Committee. A549 xenografts were established, and RBM5 gene was delivered into xenografts by attenuated Salmonella according to previous studies [18, 31]. Briefly, BALB/c athymic nude female mice (nu/nu; 4–5 weeks old) were purchased from the Institute of Zoology, Chinese Academy of Sciences (Beijing, China). A549 cells (1 × 10^7^) were suspended in 100 μl PBS and injected subcutaneously into the right flank region of nude mice.

Competent *Salmonella enterica serovar Typhimurium  * cells (competence) were mixed with 1 μg GV287-RBM5 or 1 μg control plasmids and cooled for 15 min on ice. And the plasmids were electrotransfected into the competence under the conditions as follows: C = 25 μF, PC = 200 Ω, V = 1.25 kV (12.5 kV/cm). Then, the recombinant attenuated salmonellae were quickly transferred into LB Ager medium for proliferation at 37 °C and stored at −80 °C.

The tumor-bearing mice were randomly divided into two groups (six mice per group) at day 21 after cell injection. The mice were treated at day 28 and 35 respectively through a tail mainline as follows: (a) control group (attenuated Salmonella carrying control plasmids) (10^8^ colony-forming units (CFU) per 50μlPBS); (b) RBM5 overexpression group (attenuated salmonella carrying GV287-RBM5 plasmids) (10^8^ CFU per 50 μl PBS). The mice were sacrificed on day 42, and the tumors were removed and fixed in formalin for immunohistochemistry analysis.

### Immunohistochemistry staining

Tumors treated with recombinant Salmonella strains carrying different plasmids were analyzed by immunohistochemistry staining as described previously [18]. Anti-human mouse RBM5 antibody was obtained from Santa Cruz Biotechnology (Santa Cruz Biotechnology, USA). Anti-human rabbit LC-3 antibody was obtained from Proteintech Group (Proteintech Group, USA). Anti-human rabbit LAMP1 antibody was obtained from Abcam (Abcam, USA). Anti-human rabbit Beclin1 antibody was obtained from Proteintech Group (Proteintech Group, USA).

### Statistical analysis

All data were presented as mean ± standard deviation (SD) for three independent experiments. Student’s *t* test was used to compare the difference between two groups (two-tailed; *P* < 0.05 was considered statistically significant). The analysis was performed using SPSS 17.0 software.

## Results

### Ectopic expression of RBM5 enhanced autophagic vacuoles formation in A549 cells

We previously reported that RBM5 overexpression induced apoptosis in human lung adenocarcinoma A549 cells [19, 20]. However, the relationship between RBM5 overexpression and autophagy, which is closely related to apoptosis, has not been elucidated. To investigate whether ectopic expression of RBM5 modulates autophagy machinery in A549 cells, A549 cells were transiently transfected with RBM5 expressing plasmid (GV287-RBM5) (RBM5 group) or negative control plasmid (control group).

**Fig. 1:**
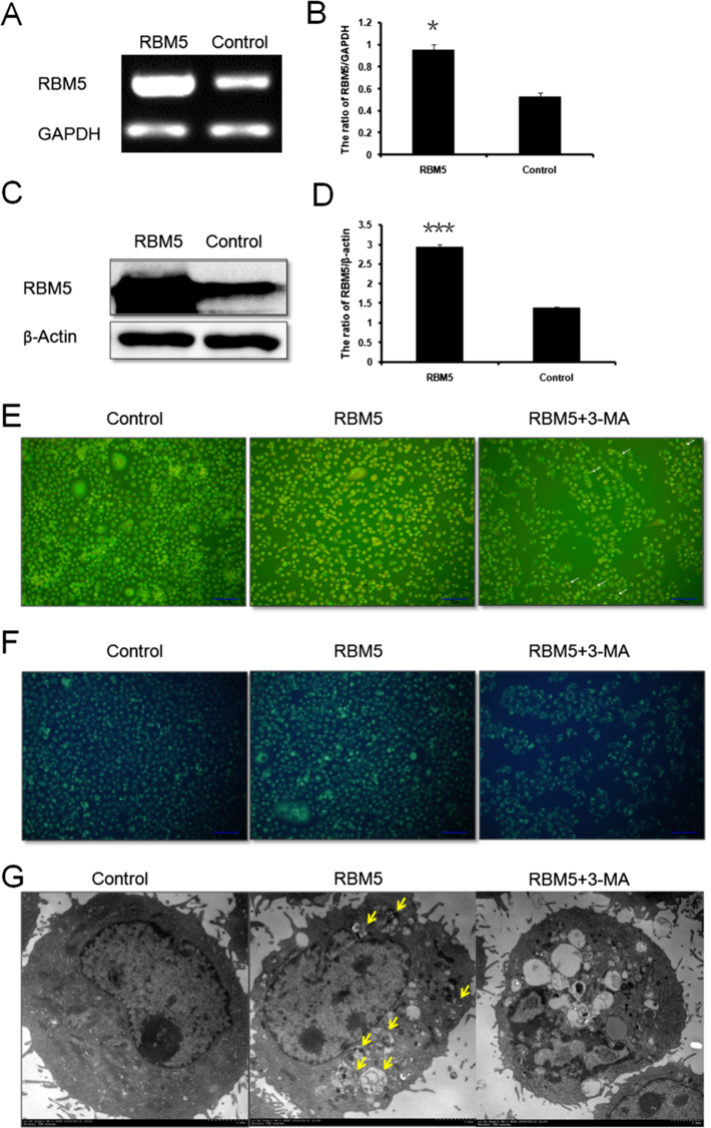
Overexpression of RBM5 enhanced autophagic vacuoles formation in A549 cells. A549 cells were transfected with recombined GV287-RBM5 plasmids (RBM5 group), plasmids with scrambled control sequence (control group), or co-treated with RBM5 transfection and classic autophagy inhibitor 3-MA (RBM5 + 3-MA group). **a** RT-PCR analysis of the mRNA levels of RBM5 and GAPDH in A549 cells. **b** Quantification of RBM5 mRNA levels relative to GAPDH. **c** Western blot analysis of the protein expression levels of RBM5 and β-actin in A549 cells. **d** Quantification of RBM5 protein expression relative to β-actin. **e** Images of acridine orange (AO) staining under fluorescence microscope. **f** Images of Monodansylcadaverine (MDC) staining. **g** Images acquired by transmission electron microscope. Data were presented as mean ± SD for three replicate experiments. *P < 0.05, ***P < 0.001

We firstly examined expression level of RBM5 by RT-PCR and Western blot analysis. The mRNA and protein expression levels of RBM5 were significantly increased in the RBM5 transfected cells (*P* < 0.05, *P* < 0.001, respectively) (Fig. 1a–d), indicating that GV287-RBM5 was successfully transfected into A549 cells.

AO and MDC are fluorescent substances, which are specific markers for autophagic vacuoles and are often used to detect the occurrence of autophagy [33]. In AO stained cells, cytoplasm and nucleolus showed green fluorescence whereas AVOs showed bright red fluorescence. Intensity of red fluorescence is proportional to the number of AVOs in autophagic cells. With AO staining, we observed that red fluorescent spots were increased in RBM5 overexpressed A549 cells compared to the control cells (Fig. 1e), indicating that RBM5 overexpression enhanced autophagic vacuoles formation. When cells were co-treated with RBM5 transfection and a classic autophagy inhibitor 3-MA (5 mM), the increment of acridine orange-positive AVOs was inhibited and there were more nucleus presenting yellow-green fluorescence by acridine orange (AO) staining and concentrating into a crescent or granules that located in one side of the cells compared to RBM5 group (Fig. 1e), suggesting that 3-MA inhibited RBM5 induced autophagy and enhanced RBM5 induced early-stage apoptosis. MDC accumulates in mature autophagic vacuoles, such as autophagolysosomes, but not in the early endosome compartment [34]. In MDC stained cells, AVOs appear as distinct dot-like structures distributed within the cytoplasm or localizing in the perinuclear regions. With MDC staining, we observed that the RBM5 overexpressed A549 cells showed a greater fluorescence intensity and a greater number of MDC-labeled particles in the cytoplasm of the cells compared to the control group (Fig. 1f), indicating that overexpression of RBM5 increased MDC recruitment to autophagosomes which was suppressed by the autophagy inhibitor, 3-MA (Fig. 1f).

Additionally, ultrastructural changes in treated cells were examined with TEM. TEM images in control group displayed normal cytoplasm and were characterized by mitochondria, endoplasmic reticulum, free ribosomes, and irregular nucleus, as well as few autophagosomes and lysosomes (Fig. 1g). In contrast, RBM5 group exhibited many autophagosomes at various stages in cytoplasm. Arrows indicated typical double-membrane-bound vacuoles containing morphologically intact cytoplasm, organelles, or electron-dense contents (Fig. 1g). The co-treated A549 cells exhibited less autophagosomes than RBM5 group but showed the following findings: peripheral chromatin condensation, undulations in the boundaries of the nucleus, lipid droplets, glycogen accumulation, mitochondrial injury, degeneration and dilatation of the endoplasmic reticulum, increased lysosomes, and intracytoplasmic vacuolization (Fig. 1g), indicating that 3-MA inhibited RBM5-induced autophagy but enhanced RBM5-induced apoptosis.

### Overexpression of RBM5 induced cell autophagy-related protein expression

**Fig. 2:**
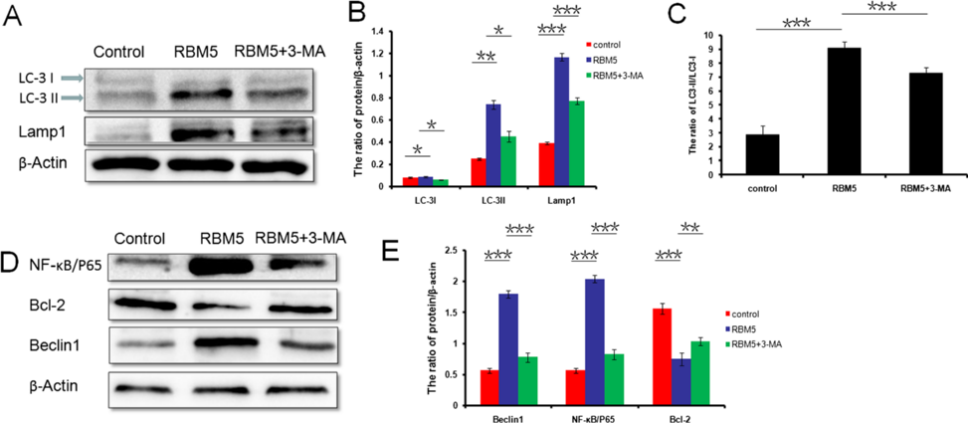
Overexpression of RBM5 induced cell autophagy-related protein expression. A549 cells were treated with RBM5 overexpression (RBM5 group), control plasmids (control group), or RBM5 overexpression combined with 3-MA (RBM5+3-MA group). **a** Western blot analysis of the protein expression levels of LC3-I, LC3-II, LAMP1, and β-actin in A549 cells. **b** Quantification of protein expression relative to β-actin. **c** Quantification of LC3-II expression relative to LC3-I. **d** Western blot analysis of the protein expression levels of NF-κB/p65, Bcl-2, Beclin1, and β-actin in A549 cells. **e** Quantification of protein expression relative to β-actin. Data shown are representative of three independent experiments and presented as mean ± SD. *P < 0.05, **P < 0.01, ***P < 0.001

To estimate whether double-membrane-bound vacuoles accumulation, which was observed in the fluorescent staining and TEM analysis, was due to an increase in the autophagic flux, we analyzed the expression levels of autophagosomal (LC3-I and LC3-II) and lysosomal (LAMP1) markers by Western blot analysis at 24 h following transfection. During autophagy, LC3-I undergoes cleavage and lipidation to yield LC3-II, which is a reliable protein marker associated with completed autophagosomes. The amount of LC3-II and the ratio of LC3-II/LC3-I might be correlated to the numbers of autophagic vesicles in the cell. The LC3-I and LC3-II expression levels (Fig. 2a, b; *P* < 0.05 and *P* < 0.01, respectively) and the ratio of LC3-II/LC3-I (Fig. 2c; *P* < 0.001) in the RBM5 group were significantly increased compared to the control group, indicating that overexpression of RBM5 induces autophagosomes formation. But the administration of 3-MA significantly decreased the expression levels of LC3-I, LC3-II (Fig. 2a, b; *P* < 0.05) and the ratio of LC3-II/LC3-I (Fig. 2c; *P* < 0.001) in the co-treatment group compared to those in the RBM5 group.

LAMP1 is a structural protein on the membranes of late endosomes and lysosomes and is well-known for its regulation of lysosomal motility and membrane fusion events between autophagosomes with late endosomes/lysosomes [35]. We observed that LAMP1 expression was increased significantly in RBM5 overexpressed A549 cells compared to that in the control group (Fig. 2a, b; *P* < 0.001), indicating an upregulation of lysosomal activity and the fusion of autophagosomes with lysosomes in the RBM5 overexpressed cells. However, 3-MA inhibited LAMP1 expression significantly in co-treatment group compared to the RBM5 group (Fig. 2a, b; *P* < 0.001).

Overall, these data indicated that RBM5 overexpression could induce autophagic flux in human lung cancer A549 cells, and this process could be suppressed by the autophagy inhibitor, 3-MA.

In order to investigate the mechanism that RBM5 induced autophagy, we evaluated the expression levels of autophagy regulators, Beclin1, NF-κB/p65, and Bcl-2 by Western blot analysis. Beclin1 plays an important role in initiating the formation of autophagosomes, NF-κB/p65 is an inducer of autophagy through the activation of Beclin1 [36], and Bcl-2 is an inhibitor of Beclin1-dependent autophagy [24]. The results showed that the expression levels of Beclin1, as well as NF-κB/p65, were increased significantly (*P* < 0.001) in RBM5 overexpression group, which were inhibited by co-treatment of RBM5 and 3-MA (Fig. 2d, e). On the contrary, the expression level of Bcl-2 was decreased significantly in RBM5 overexpression group compared to the control group (Fig. 2d, e; *P* < 0.001) and can be reversed by 3-MA in the co-treatment group (Fig. 2d, e; *P* < 0.01). These data suggested that overexpression of RBM5 could induce autophagy through the upregulation of Beclin1, which might consequently switch on the downstream reaction via the activation of NF-κB/p65,inactivation of Bcl-2, and invoke the autophagy process.

### Pharmacological modulation of autophagy enhanced overexpressed RBM5-induced apoptosis and chemosensitivity

**Fig. 3:**
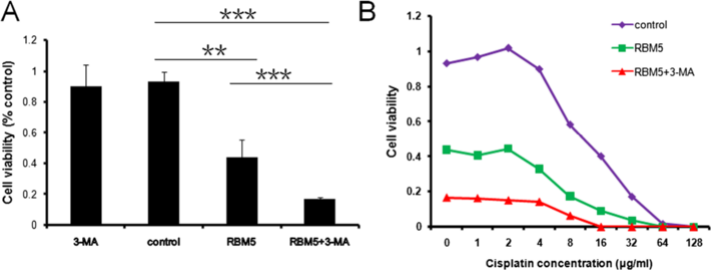
Pharmacological modulation of autophagy enhances overexpressed RBM5-induced apoptosis. A549 cells were treated with RBM5 overexpression (RBM5 group), control plasmids (control group), RBM5 overexpression combined with 3-MA (RBM5 + 3-MA group), or 3-MA alone (3-MA group). **a** Cell viability was examined by MTT assay. **b** Transfected A549 cells were treated with varying concentrations of cisplatin for 24 h. Cell viability was determined by MTT assay. Data were presented as mean ± SD for three replicate experiments. **P < 0.01, ***P < 0.001

To determine whether autophagy induced by RBM5 overexpression played a cytoprotective or cytotoxic role in A549 cells, we applied autophagy inhibitor 3-MA to suppress autophagy and examined cell viability by MTT assay. Our previous studies showed that RBM5 overexpression inhibited the growth of both A549 and A549/DDP cells and sensitized both cells to cisplatin [19, 20]. Similarly, RBM5 overexpressed A549 cells showed a significantly lower survival rate (Fig. 3a) and increased sensitivity to cisplatin-induced growth inhibition (Fig. 3b) compared to control group. While treated with 3-MA alone had a slightly decrease but not a significant effect on cellular proliferation, co-treatment with 3-MA and RBM5 transfection significantly decreased cell viability (Fig. 3a) and increased sensitivity to cisplatin-induced growth inhibition (Fig. 3b) compared to the cells treated with RBM5 transfection alone.

Our previous studies showed that RBM5 inhibited the growth of A549 cells and resensitized A549/DDP cells to cisplatin through apoptosis [8, 18–20]. In the present study, we found that inhibition of autophagy, which was induced by the co-treatment of overexpressed RBM5 and 3-MA, enhanced RBM5 induced A549 cell growth inhibition and sensitivity to cisplatin. The data above suggested that autophagy activated by RBM5 overexpression functions as a pro-survival factor and prevents cell death triggered by RBM5. And autophagy inhibition by 3-MA could enhance RBM5-induced apoptosis and chemosensitivity.

### RBM5 overexpression induced autophagy in A549 xenografts in BALB/c nude mice

**Fig. 4:**
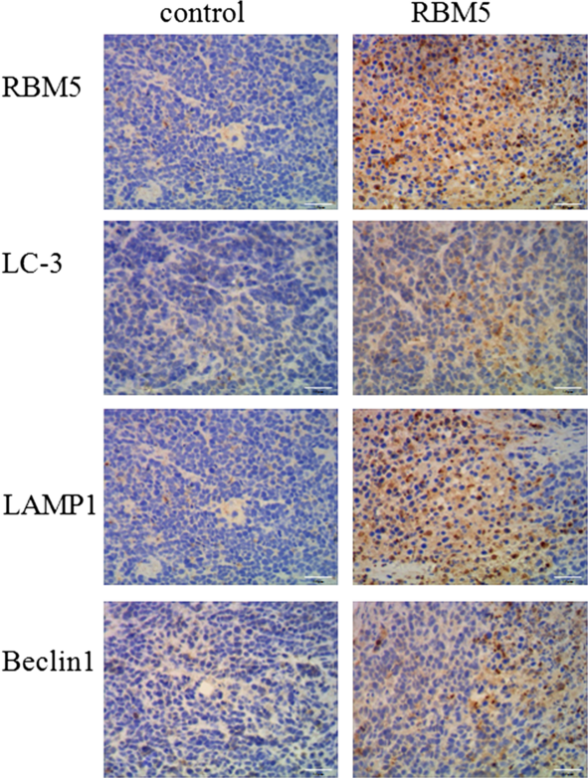
Immunohistochemistry assessment of RBM5 and candidate markers of autophagy in A549 xenograft. A549 xenografts were established and treated with attenuated Salmonella carrying control plasmids (control group) or GV287-RBM5 plasmids (RBM5 group). The mice were sacrificed on day 42 and the tumors were removed. Expression levels of RBM5 and candidate markers of autophagy, LC3, LAMP1, and Beclin1, were assessed by immunohistochemistry staining

To determine the potential impact of RBM5 overexpression on lung cancer cells in vivo, A549 xenografts were treated with control plasmid or GV287-RBM5 plasmid via attenuated Salmonella. It has been confirmed that RBM5 overexpression contributed to the retardation of the tumor growth and cell death in A549 xenografts in previous studies [18, 31]. To further validate the autophagy induced by RBM5 overexpression, we assessed the expression levels of RBM5 and candidate markers of autophagy, LC3, LAMP1, and Beclin1, by immunohistochemistry staining in the tissue from the tumor xenografts in our in vivo studies following treatment with attenuated Salmonella carrying control plasmids or GV287-RBM5 plasmids. First, overexpression of RBM5 was confirmed by immunohistochemistry staining. As shown in Fig. 4, RBM5 expression was significantly higher in RBM5 group compared to the control group, suggesting that the RBM5 was efficiently delivered into tumor xenografts and overexpressed by attenuated Salmonella in vivo. In agreement with above cell culture findings, RBM5 overexpression in tumor xenografts induced LC-3 and LAMP1 localization, indicating that RBM5 induced autophagic flux in vivo. Moreover, Beclin1 expression was significantly higher in RBM5 group compared to the control group, suggesting that the RBM5 induced autophagy through activation of Beclin1.

## Discussion

Previous studies demonstrated that RBM5 displayed effective antiproliferative activity in cell lines derived from human solid tumors, such as lung cancer and prostate cancer, including multidrug resistant cells [8, 18–20]. RBM5 is known as a tumor suppressor gene that has been shown to function on cell growth inhibition by inducing apoptosis. Autophagy and apoptosis are closely related programmed cell activities, which are of great importance to determine cell death and/or survival. Autophagy is emerging as a key process modulating tumorigenesis, tumor-stroma interactions and cancer therapy, but plays a dual, context-dependent role in tumor suppression and survival [37]. Autophagy plays a protective role in cancer cells in certain circumstances [38]; however, in other circumstances it is involved in type II programmed cell death [39, 40]. From a therapeutic perspective, understanding when and how autophagy can be harnessed to kill cancer cells remains challenging [41]. Therefore, a better understanding about regulation relationship of RBM5 on autophagy and the role autophagy plays in cell survival is critical for developing therapeutic strategies targeting RBM5 in lung cancer and understanding more about mechanisms of autophagy regulation.

In the present study, we experimentally conducted overexpression of RBM5 in human lung adenocarcinoma A549 cells and for the first time demonstrated that autophagy, aside from apoptosis alone, was also activated by RBM5 overexpression. The increased autophagic flux in the RBM5 overexpressed A549 cells was demonstrated by the enhancement of LC3-II, LC3-II/LC3-I, and Beclin1 together with the accumulation of AVOs assessed by AO staining, MDC staining and TEM, and the upregulation of lysosomal activity and autophagolysosomal fusion characterized by increased levels of LAMP1. In order to confirm our in vitro results, the lung adenocarcinoma transplantation in vivo model was made by injecting A549 cells onto the back of immunocompromised mice and RBM5 gene was delivered into xenografts by attenuated Salmonella. As shown in the results, increased autophagic flux in the RBM5 overexpressed A549 xenografts was confirmed by the overexpression of LC3, Beclin1, and LAMP1 evaluated by immunohistochemistry staining.

Autophagy induction in mammalian cells is mainly dependent upon the activation of the class III PI3K or PI3K/AKT/mTOR pathways [42]. Beclin1 (Atg6) is a well-known key regulator of autophagy [43], which governs the autophagic process by regulating class III PI3K pathway activated autophagosome formation [44, 45]. Increased Beclin1 expression was also observed in the RBM5 overexpressed A549 cells and A549 xenografts in the present study. However, when Beclin1-PI3K III complex was inhibited by 3-MA, Beclin1 expression was downregulated and autophagic flux was decreased, confirming a crucial role for Beclin1 in RBM5-induced autophagy in lung cancer cells.

Bcl-2, an anti-apoptotic protein, is also a Beclin1 binding protein and act as an autophagy regulator [22–24]. As demonstrated in previous studies [18, 19], we found that RBM5 overexpression decreased the level of bcl-2 protein that occurred, along with the induction of autophagy in A549 cells in the present study, indicating that downregulation of Bcl-2 was also involved in RBM5-induced autophagy. Interestingly, we also found that administration of 3-MA prevented RBM5-induced downregulation of Bcl-2. Likewise, results were reported by Xie H et al. that Beclin1 proteins was decreased but Bcl-2 was upregulated in 3-MA-treated H9c2 rat cardiomyocytes [46], which might be due to strong interaction between Beclin1 and Bcl-2.

NF-κB/p65 is another regulator of Beclin1-mediated autophagy [36, 47], which could be inhibited by 3-MA [48]. Our results again showed that overexpression of RBM5 activated Beclin1-mediated autophagy mainly through activation of NF-κB/p65 pathway and inhibition of Bcl-2. NF-κB/p65 protein levels, along with cell autophagy were overexpressed by RBM5 overexpression, which was inhibited by 3-MA in A549 cells, indicating that NF-κB/p65 was regulated by RBM5 and was involved in RBM5-induced autophagy.

However, the mechanisms that RBM5 regulates Beclin-1, Bcl-2, and NF-κB/p65 still need to be investigated. It is reported that RBM5 is a component of prespliceosomal complexes that regulates the alternative splicing of several mRNAs, such as Fas and caspase-2 [49, 50]. As overexpression of Beclin1 and NF-κB/p65 and downregulation of Bcl-2 can be inhibited by 3-MA, RBM5 may directly or indirectly regulate those genes expression through regulating such genes expression of class III PI3K pathway, like Beclin1 or other genes, by alternative splicing of mRNAs.

Besides those genes examined in this study, our previous studies revealed that RBM5 also regulates such genes expression as Bcl-2 family [18, 19], EGFR [4, 31], and β-catenin [51], which are all important autophagy regulators [23, 24, 52–55]. Thus, it is a complicated process of autophagy regulation by RBM5 that needs to be further investigated.

The role of autophagy in carcinogenesis is a paradox, which can be a pro-survival mechanism to deteriorate therapeutic outcomes or act as programmed cell death to improve overall anti-tumor efficacy [56]. In order to investigate the role of activated autophagy plays in RBM5-induced cell death, the present study used a specific autophagy inhibitor, 3-MA, which is a class III phosphatidylinositol 3-kinase (PI3K) inhibitor. RBM5 overexpression induced autophagy in A549 cells was significantly inhibited by the administration of 3-MA. Furthermore, the inhibition of autophagy enhanced RBM5-induced cell death and increased cancer cell sensitivity to cisplatin in A549 cells, maybe through enhanced apoptosis. These findings indicate that autophagy may be utilized as a protective mechanism against cell death in RBM5 overexpressed A549 cells and that its inhibition may improve the anti-tumor efficacy of RBM5 in lung cancer therapies.

Apoptosis is a natural way of removing aged cells from the body and is the way by which most of the anti-cancer therapies eliminate malignant cells. However, several pathways modulate the apoptosis signaling and contribute to apoptosis resistance in cancers, such as Bcl-2 and Mcl-1 proteins, autophagy processes, aberrant nuclear export signaling, etc [57]. Autophagy has been observed to protect cancer cells from apoptosis upon certain anti-cancer drugs [58, 59] and gene targeting therapies [60, 61]. Our previous studies demonstrated that RBM5 can inhibit the growth of lung cancer cells and enhance cancer cells’ sensitivity to cisplatin through apoptosis [8, 18–20]. In the present study, we found that autophagy induced by RBM5 overexpression in lung cancer cells acted a pro-survival way. However, actually RBM5 overexpression caused cell growth inhibition, indicating that in the lung cancer cells, RBM5-induced apoptosis overweighs RBM5-induced autophagy and induces apoptotic cell death. Co-treatment with 3-MA inhibited RBM5-induced autophagy, enhanced RBM5-induced apoptosis and chemosensitivity. Thus, modulating the sensitivity and regulatory mechanism of cells to RBM5 overexpression, such as through the use of 3-MA, could potentially serve as a strategy in sensitizing and reducing resistance of lung cancer cells to RBM5 targeting therapy by blocking RBM5-mediated protective autophagy.

## Conclusions

Overall, our data revealed that the pro-survival autophagy is enhanced by ectopic RBM5 overexpression, and inhibition of autophagy can enhance RBM5-induced apoptosis and increase cancer cells sensitivity to cisplatin. Our study suggests that a combination of RBM5 overexpression and autophagic inhibitor could be a good approach to treat NSCLC and resensitize cells to anti-tumor drugs.
